# A Multidisciplinary Perspective on Breast Phyllodes Tumors: A Literature Review

**DOI:** 10.3390/medicina61101883

**Published:** 2025-10-21

**Authors:** Alexandru-Gratian Naum, Andra-Mara Ursu, Paloma Moisii, Corina-Veronica Lupascu-Ursulescu, Liliana Gheorghe, Irina Jari

**Affiliations:** 1 Department of Morpho-Functional Sciences (II), Grigore T. Popa University of Medicine and Pharmacy Iasi, 700115 Iasi, Romania; 2 Individual Medical Practice, 700399 Iasi, Romania; 3 Department of Medical Specialties (I), Grigore T. Popa University of Medicine and Pharmacy Iasi, 700115 Iasi, Romania; paloma.manea@umfiasi.ro; 4 Department of Surgery (II), Grigore T. Popa University of Medicine and Pharmacy Iasi, 700115 Iasi, Romania; corina.ursulescu@umfiasi.ro (C.-V.L.-U.); liliana.gheorghe@umfiasi.ro (L.G.); irina.jari@umfiasi.ro (I.J.)

**Keywords:** phyllodes tumor, breast, benign, borderline, malignant, ultrasound, mammography, MRI

## Abstract

Phyllodes tumors, also known as cystosarcoma phyllodes, represent a rare and complex category of fibroepithelial neoplasms that primarily affect the breast. These tumors are characterized by their unique histological architecture, which resembles leaf-like structures, as suggested by the etymology of the term “phyllodes,” derived from the Greek word “phyllodes,” meaning “leaf-like”. The World Health Organization (WHO) has classified these tumors into three distinct categories—benign, borderline, and malignant—based on various histopathological criteria, including cellular atypia, mitotic activity, and stromal overgrowth. With a peak incidence occurring between the ages of 40 and 52, these tumors primarily affect women and constitute 0.3% to 1% of all breast tumors. Imaging modalities currently employed (mammography, ultrasound, and MRI) play a crucial role in the initial assessment of breast masses. Histopathological characteristics, such as stromal cellularity and mitotic activity, and immunohistochemical markers, like Ki-67 and p53, are important in the diagnosis, categorization, treatment plans, and prognosis of breast phyllodes tumors. Surgical intervention, with the goal of achieving complete excision of the tumor along with adequate margins, is the primary treatment option. Adjuvant therapies, such as radiotherapy, may be considered but are still debatable. Understanding the nuances of these tumors is crucial for healthcare professionals, as they present unique challenges in both diagnosis and treatment.

## 1. Introduction

Phyllodes tumors, which are also called cystosarcoma phyllodes, are an uncommon and varied type of fibroepithelial neoplasms that mostly show up in the breast but can also occur in the skin, inside the digestive tract, or even in the reproductive organs. These tumors, initially described in 1838, are characterized by their distinctive histological features, which include stromal overgrowth and a leaf-like architecture, hence the name “phyllodes” derived from the Greek words phyllos (leaf) and eidos (form). Phyllodes tumors can behave in a wide range of ways, from being harmless to cancerous. Because of this, it is important to fully understand their pathophysiology, diagnosis, and treatment options [1,2]. Cystosarcoma phyllodes and periductal stromal sarcoma are historical synonyms for the phyllodes tumor (PT). This tumor has been found sometimes in the prostate and seminal vesicles, but its main location is in the female breast, where it constitutes about 0.3% to 1.0% of all primary breast tumors and 2% to 3% of fibroepithelial lesions in Western countries [1,3,4,5]. The projected yearly incidence of these tumors is around 2.1 cases per million women, constituting 0.3 to 1% of all breast tumors in women. Although they can occur at any age, phyllodes tumors are most frequently diagnosed in women aged 35 to 55, with a peak incidence between 40 and 50 years [6,7,8]. Notably, these tumors are exceedingly rare in men, with only a handful of cases documented in the literature [9]. Furthermore, epidemiological studies indicate that Latina, Hispanic, and Asian women exhibit a higher incidence of phyllodes tumors compared to their Caucasian counterparts. Interestingly, the age distribution of these tumors follows an ethnic preference pattern, with Asian women being diagnosed at a younger age than Caucasians [5,6,8].

WHO classifies PTs according to their pathological characteristics into three categories—benign (B), borderline (BL), and malignant (M) tumors—the latter of which includes sarcoma phyllodes, noted for a high risk of recurrence. Based on imaging characteristics, clinical presentation, and growth rate, PT may resemble a fibroadenoma (FA) or a breast carcinoma (BC). The histological grade of PT correlates with patient age, with higher-grade tumors being more frequently observed in older patients [2].

The literature cites the coexistence of PT with other tumors such as BC [3]. Understanding the nuances of these tumors is crucial for healthcare professionals, as they present unique challenges in both diagnosis and treatment [10,11,12]. This review aims to explore the complexities surrounding phyllodes tumors, including their pathophysiology, histological features, and the critical role of immunohistochemical markers in diagnosis and treatment.

## 2. Methods

**Figure 1:**
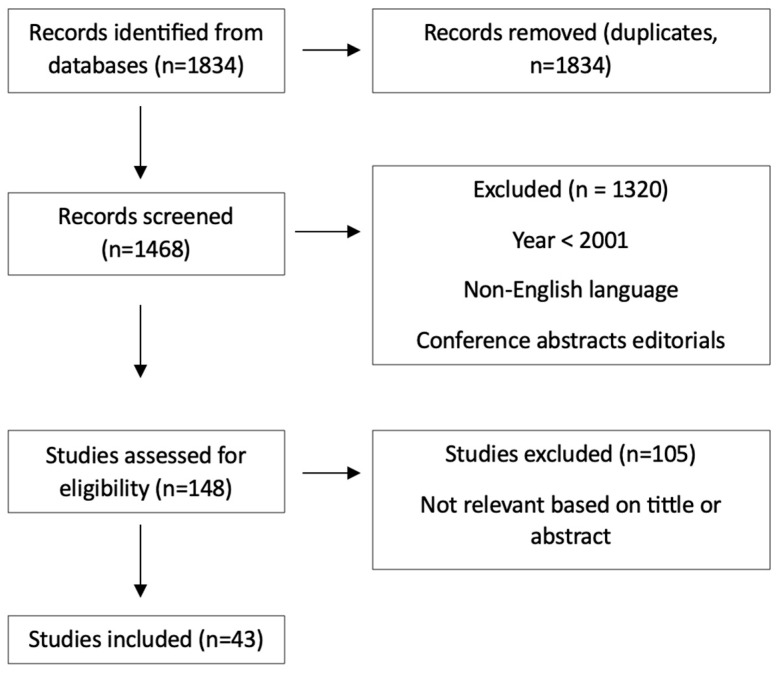
Prisma diagram for articles’ including/excluding criteria.

The keywords “Cystosarcoma phyllodes,” and “Phyllodes Tumor” were used to conduct a search on Medline, PubMed, and Web of Science databases. A total of 1834 references were identified that were published between 1 January 2013, and 30 June 2025. Non-English language papers, reviews, conference abstracts, editorials, commentaries and correspondence were excluded (*n* = 1646). Articles that were not pertinent to the subject matter were excluded by reviewing their abstracts and titles (*n* = 148). The full texts of 40 articles that were potentially relevant were retrieved and evaluated (Figure 1).

## 3. Clinical Presentation and Diagnosis

PTs predominantly affect women, although a few sporadic cases have been reported in men, appearing at younger ages compared to women (25–30 years). Dzulhijar et al. [9] conclude that PTs in men may appear associated with gynecomastia, prostate conditions, potentially due to estrogen–androgen imbalance and after hormone therapy for prostate cancer.

Patients with PTs often present with a palpable breast mass [4] that may be associated with local symptoms such as pain or tenderness. The rapid growth of these tumors can lead to significant anxiety among patients, prompting them to seek medical evaluation [13]. Clinical examination typically reveals a well-defined, mobile mass that may vary in size from a few centimeters to several decimeters. Jagdewsing et al. [14] reported an average tumor size of 8.3 ± 5.8 cm, in contrast to mean dimensions (MD) and range (R) noted in other studies: 4–5 cm (Yamada et al., cited by Lu.) [15] or 30.6 ± 28.6 cm (Di Liso et al.) [4] (Table 1). The occurrence of rapid mass growth varies significantly among different studies: reported rates include 12 to 51.7% [5,7], while some authors (Di Liso, Jagdewsing, and Lin) did not address this aspect at all [4,14,16]. Skin changes or breast contour distortion may accompany the mass in some cases, further complicating the clinical picture [5].

No cohort studies are available for PTs in men so the incidence in the case of men is not in fact known. The majority of the studies are conducted on women so PT incidence refers mainly to the incidence among women, and each study covers different countries and variable periods of time. Different authors cite an overall incidence of PTs: 0.3–1.0% of all primary breast tumors [9,10]. Most of the data concerns the U.S.A, with an average incidence of 2.1 per year per million women. In Los Angeles County, the average yearly incidence rate of M PTs over a 17-year period was 2.1 per million women with a lower incidence of PTs in non-Latina white, Asian, and African American women. Other authors referred to the incidence of PTs among FAs (2.5% in Western countries and 1.7–19.6% in Japan) with a ratio of M PTs between 9.2% and 51% in Western countries, and between 2.8% and 38% in Asia. Fukami et al. suspect that the different percentages between Western European countries and Asian countries are due to hospital diagnostic criteria and not due to racial difference [4,8,13].

Concerning patient’s age, the data are heterogeneous among different authors as follows:

- Katiwada (USA) stated, studying the literature, that PTs occur in women with a median age of 45–49 years; age of appearance of PTs was earlier among people of Asian and Latino-White origin from USA, and more frequent recurrence was observed [8].
- Some of the Japanese authors stated that the patients diagnosed with PTs are younger in Japan, (23–35 years) and older in Western countries (40 years old), but Inoshita reported that the patient’s mean age was 43 years old. Patients with M PTs had a mean age of 43.9 years, with no M or BL tumors in case of teenagers in Japan [11]. Western countries reported teenagers with M PTs [4].
- Studies conducted in China had concordant results: most BPTs were found among younger women, while BL and M variants were more frequent among elderly patients (mean age of 46–47 years) [5,7].
- Chin Chan Lin’s study of 33 Asian patients from Taiwan found the same mean age at diagnosis as other populations (49 years old), and confirmed a higher recurrence rate (27% compared to 21% in the WHO report) [16].

Imaging studies, including mammography and ultrasound, play a crucial role in the initial assessment of breast masses. The diagnostic algorithm is age-dependent: mammography is utilized for women over 40–45 years, while breast ultrasound is employed for those under 40, often in conjunction with an MRI and core needle biopsy. The imaging features are not enough to clearly tell the difference between PTs, FAs, and breast cancer, or between the different types of PTs [17].

Mammography reveals a large mass that may be regular or lobulated, typically characterized by smooth margins, although some cases may exhibit partially indistinct or irregular margins. Moreover, one may observe features like a radiolucent halo, calcifications, or diffuse skin thickening, especially in MPTs [14].

Ultrasound imaging may show a mass that resembles a follicular adenoma; however, hypoechoic solid masses exhibiting posterior acoustic enhancement are more characteristic of pleomorphic adenomas. Features such as an inhomogeneous structure with cystic spaces, thick septa, microlobulated margins, and significant vascularity raise the likelihood of a PT diagnosis and increase the suspicion of malignancy [8,18].

PTs present as masses with heterogeneous enhancement on MRI. BPTs generally exhibit low signal intensity on T1-weighted images and high signal intensity on T2-weighted images, characterized by a homogeneous structure and contrast enhancement. MPTs exhibit a heterogeneous structure and varying signal intensity on T1- and T2-weighted images, characterized by cystic spaces and thick, irregular septa with intense enhancement, and may also present hyperintense regions on T1 attributed to hemorrhage. In dynamic postcontrast imaging, the initial segment of the enhancement curve is rapid for MPTs. The presence of restricted diffusion is a sign of malignant transformation. The malignant PT shows a high signal on (DWI), but a low signal on the apparent diffusion coefficient (ADC) [18,19].

Computed tomography (CT) scans are not used very often, but when they are, they show dense masses or groups of multiple soft tissue density masses that are clearly defined and show different levels of enhancement [15].

## 4. Histopathological Features

Integrating imaging techniques with histopathological analysis is the best for the diagnosis and treatment of breast tumors, including PTs. Histologically, PT is characterized by a fibroepithelial composition, exhibiting an irregularly expansive, cellular proliferation of specialized stroma. The tumors display a prominent leaf-like architecture, which serves as a distinguishing feature from fibroadenomas. Phyllodes tumors usually have a higher stromal cellularity density and a lot of stromal nuclear heterogeneity [20,21]. This makes it important to look at the stromal features to tell PTs apart from other fibroepithelial lesions. A WHO classification system for phyllodes tumors looks at five factors: the number of cells in the stroma, the activity of mitoses, the shape of the cells, the amount of stromal overgrowth, and the edges of the tumor. The most common histological type is benign, accounting for 60% to 78% of cases, while borderline and malignant tumors comprise 6% to 35% of lesions, respectively. Understanding these histopathological features is essential for pathologists and oncologists in determining the appropriate management strategies for affected patients. Histology is crucial for diagnosing and prognosticating PT. Some tumors may sometimes grow inside FAs. PTs have a hypercellular stroma, cleft-like spaces, and a leaf-shaped pattern [22,23].

All authors categorized breast PTs into three groups (B, BL, and M tumors), using the WHO criteria 2019 [4].

BPTs exhibit fewer than 3 mitoses per 10 high-power fields (HPF) and display mild stromal atypia. In contrast, BPTs present with 4–9 mitoses per 10 HPF, while M PTs demonstrate more than 10 mitoses per HPF along with necrosis.

Zhi-Rui Zhou classified BPTs as a low-risk group and BL and MPTs as a high-risk group. Zhou classified approximately 75% of PTs as B, 16% as BL, and 9% as M [7]. This classification system, adopted in 1982 and updated in subsequent years, highlights the importance of accurate diagnosis and management strategies for phyllodes tumors [2].

## 5. Immunohistochemical Markers (IHC)

Various markers have been employed to elucidate the cellular composition and biological behavior of these tumors.

Estrogen Receptor (ER) and Progesterone Receptor (PR). Some benign phyllodes tumors have low levels of ER and PR expression, while some malignant variants may have different levels of receptor positivity. Checking the ER and PR status can help us understand how these tumors react to hormones and could change how we treat them, especially when we think about adding hormone therapy on top of the main treatment. Also, the fact that hormone receptors are present may mean that targeted therapies are possible, which could help treat more aggressive types of phyllodes tumors [23].

Ki-67 Proliferation Index. In phyllodes tumors, a high Ki-67 proliferation index is indicative of a malignant phenotype and is associated with a poorer prognosis. Consequently, the evaluation of Ki-67 expression can aid in stratifying patients based on their risk of recurrence and metastasis, guiding clinical decision-making. A higher Ki-67 index often necessitates more aggressive treatment approaches, including surgical intervention and close follow-up. The Ki-67 marker is particularly useful in borderline tumors, where distinguishing between benign and malignant behavior can be challenging. Torres et al. identified a significant correlation between Ki-67 expression and BL tumors. This indicates that Ki-67 could serve as a valuable instrument in the classification of PT, particularly in complex cases. Studies indicate that Ki-67 does not consistently correlate with tumor recurrence; thus, while it may offer insights into tumor aggressiveness, it should not be the sole predictor of recurrence in PTs [24].

P53 Tumor Suppressor Protein. Aberrant expression of p53 has been linked to malignant transformation. The presence of p53 overexpression, often assessed through immunohistochemistry, may serve as a prognostic marker, with its positivity correlating with a higher likelihood of aggressive behavior and adverse clinical outcomes. The evaluation of p53 status can provide valuable information regarding the tumor’s potential for metastasis and recurrence. Furthermore, understanding the role of p53 in PTs may open avenues for targeted therapies aimed at restoring normal p53 function or exploiting its pathways for therapeutic benefit [24].

CD117 (c-KIT). The expression of CD117 in phyllodes tumors has been documented, with some studies suggesting that its presence may be associated with malignant variants. The expression of CD117 may serve as a biomarker for identifying tumors that are more likely to respond to specific therapies, thereby personalizing treatment approaches. Understanding the role of CD117 in the biology of phyllodes tumors could lead to improved management strategies and better patient outcomes. In comparison to 90% of MPTs, merely 40% of BPTs exhibit positive results for CD117 (c-kit), a trans-membrane protein receptor encoded by the c-kit proto-oncogene, or CD34, which is associated with stem cell factor, a crucial hematopoietic regulator [25,26].

Vimentin. In phyllodes tumors, vimentin expression is typically strong, reflecting the stromal component of these neoplasms. The assessment of vimentin can aid in distinguishing phyllodes tumors from other breast lesions, particularly in cases where the histological features are ambiguous. Vimentin positivity is often associated with the aggressive behavior of tumors, making it a valuable marker in the diagnostic process. Moreover, vimentin’s role in epithelial–mesenchymal transition suggests that its expression may be linked to the invasive potential of phyllodes tumors. Understanding the implications of vimentin expression could provide insights into the mechanisms underlying tumor progression and metastasis [22].

Smooth Muscle Actin (SMA). Smooth muscle actin can be utilized to characterize the myofibroblastic component of phyllodes tumors. The presence of SMA positivity is often indicative of the myofibroblastic differentiation within the stromal component, which can further elucidate the tumor’s biological behavior. The evaluation of SMA expression can also assist in differentiating phyllodes tumors from other soft tissue tumors, particularly those with overlapping histological features. SMA expression may also correlate with the tumor’s aggressiveness, providing additional prognostic information [22].

Immunohistochemical markers, including Ki-67, p53, and CD117 (c-kit), evaluate the biological activity of PTs [13]. Torres et al. identified a significant correlation between Ki-67 expression and BLPT in comparison to BPT, as well as a notable distinction between M and BL tumors. This indicates that Ki-67 could serve as a valuable instrument in the classification of PT, particularly in complex cases. Studies indicate that Ki-67 does not consistently correlate with tumor recurrence; thus, while it may offer insights into tumor aggressiveness, it should not be the sole predictor of recurrence in PTs [18].

Reduced *Bcl-2* levels (protein that blocks programmed cell death) indicate aggressive tumors, and increased *CD10* (cell membrane zinc-dependent metalloendopeptidase) appears in malignant lesions. Vascular endothelial growth factor (VEGF) stimulates angiogenesis and tumor growth [20].

Spindle cell neoplasms and metaplastic carcinomas represent the two primary differential diagnoses for MPT. The diagnosis of MPTs presents challenges due to diffuse stromal overgrowth and the absence of CD34 expression, which may resemble metaplastic carcinoma, particularly in core biopsies. The differentiation of MPT from metaplastic carcinoma carries significant clinical implications, particularly regarding treatment modalities such as surgery, chemotherapy, and radiation therapy [23,26].

**Figure 2:**
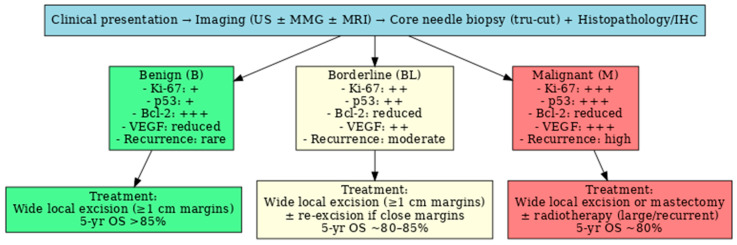
Therapeutic guidelines for benign, borderline and malignant PhTs in correlation with immunohistochemical markers (the symbols “+”, “++”, and “+++” indicate the semi-quantitative level of immunohistochemical expression low, moderate, and high, respectively for each marker, as routinely used in pathology reports).

Therapeutic guidelines for benign, borderline and malignant PhTs, in correlation with immunohistochemical markers are presented in Figure 2.

## 6. Treatment Strategies

The management of phyllodes tumors primarily involves surgical intervention, with the goal of achieving complete excision of the tumor along with adequate margins. The surgical approach may vary depending on the tumor’s size, location, and histological classification. The National Comprehensive Cancer Network Guidelines recommends wide excision without axillary staging, emphasizing that in M or BL cases, wide excision means excision with the intention of obtaining margins of >1 cm. In some cases, mastectomy may be warranted, particularly for larger tumors or those with aggressive histological features. The ideal surgical margin width for phyllodes tumors remains a topic of ongoing debate, with recommendations ranging from 1 cm to greater than 2 cm. Studies have shown that positive surgical margins are associated with an increased risk of local recurrence, underscoring the importance of achieving clear margins during surgical resection [27,28]. In cases where negative margins cannot be achieved, adjuvant therapies such as radiotherapy may be considered, particularly for malignant phyllodes tumors. Radiotherapy has been suggested as an adjunctive treatment to improve local control and reduce recurrence rates. However, the evidence supporting its efficacy remains conflicting, with some studies indicating a potential benefit in patients with borderline and malignant phyllodes tumors, while others report no significant impact on outcomes [29,30,31,32]. Consequently, the role of adjuvant radiotherapy in the management of phyllodes tumors continues to be a subject of investigation. Ongoing clinical trials are exploring the optimal timing, dosage, and patient selection for radiotherapy in this context [33,34]. Most studies concluded that all PTs can recur, including BPTs, which in some cases, progress to a more aggressive form, including MPT lesions, but only BL and MPTs tend to hematogenous metastasize to the lung, pleura, bone and even pancreas or skin [35,36,37,38].

Adjuvant radiotherapy (RT) to the whole breast (50 Gy/25 Fx) after breast-conserving surgery or to the chest wall (50 Gy/25 Fx) after mastectomy can be used [33].

The efficiency of adjuvant RT for PT remains uncertain, so RT has to be recommended taking into account the histological type, surgical approach, tumor border, and tumor residue. The adjuvant RT efficiency was demonstrated by several studies which depicted the following situations [34,39,40,41]:

- reduces the recurrence rate of BL and M PTs treated by breast-conserving surgery with negative surgical margins [34,39];
- reduces the recurrence rate in cases with a tumor size > 2 cm undergoing local excision and those with a tumor size > 10 cm undergoing mastectomy [40];
- a higher 5-year-survival rate in M PTs [41].;
- improved 10-year-local control rate of PT in BL and M groups without affecting overall survival, or without benefiting disease-free survival [34].

## 7. Prognostic Factors and Recurrence Risk

The prognosis for patients with phyllodes tumors is influenced by several factors, including tumor size, histological classification, and surgical margin status. Studies have demonstrated that larger tumors and those classified as malignant are associated with a higher risk of local recurrence and distant metastasis. The local recurrence rate for benign phyllodes tumors is relatively low, typically ranging from 10% to 20%, while borderline and malignant tumors exhibit recurrence rates of 14% to 36%; a comparison of recurrence among different studies is indicated in Table 2. Di Liso reported a recurrence rate of 8.1% in BPTs compared to 6.7% in BL PTs, while M PTs had the highest recurrence rate at 20.0%. However, the differences across these categories did not reach statistical significance (*p* = 0.212), and the comparison between B/BL PTs and M PT showed weak significance (*p* = 0.081) [4]. The identification of clinicopathological factors that predict recurrence is essential for guiding treatment decisions and follow-up strategies. Factors such as tumor size, histological grade, and margin status have been shown to correlate with recurrence risk. For instance, a comprehensive analysis of phyllodes tumors indicated that positive surgical margins and larger tumor size are significant predictors of local recurrence [24]. Furthermore, the presence of stromal overgrowth and high mitotic activity are also associated with poorer outcomes. Additionally, the biological behavior of borderline phyllodes tumors poses unique challenges, as they exhibit characteristics that straddle the line between benign and malignant. Genetic profiling and molecular studies may provide insights into the behavior of these tumors and help identify patients who may benefit from more aggressive treatment strategies [42].

## 8. Future Perspectives and Research Directions

Given the rarity of phyllodes tumors and the complexities associated with their management, continued research is essential to enhance understanding of their biology, improve diagnostic accuracy, and refine treatment strategies. Future studies should focus on elucidating the molecular and genetic underpinnings of phyllodes tumors, as well as identifying potential biomarkers that may aid in predicting clinical outcomes. Advances in genomic sequencing technologies may facilitate the discovery of novel therapeutic targets and improve risk stratification for patients. Additionally, the exploration of novel therapeutic approaches, including targeted therapies and immunotherapy, may hold promise for improving outcomes in patients with malignant phyllodes tumors. Collaborative efforts among multidisciplinary teams, including surgical oncologists, medical oncologists, radiologists, nuclear medicine specialists, and pathologists, will be crucial in advancing the field and optimizing patient care [43]. The establishment of tumor registries and collaborative research networks may also enhance data collection and facilitate large-scale studies to better understand the epidemiology and treatment outcomes of phyllodes tumors. Their complex histopathological features, variable clinical behavior, and potential for recurrence necessitate a comprehensive understanding of their diagnosis, treatment, and follow-up strategies. As research continues to evolve, the hope is to improve patient outcomes and establish more standardized management protocols for this rare tumor type. The integration of emerging technologies and collaborative research efforts will be pivotal in addressing the current gaps in knowledge and enhancing the care of patients affected by phyllodes tumors.

We studied in the literature further research directions concerning other immunohistochemical markers and discovered that MED12 controls hematopoiesis and H3K27me3 plays a pivotal role in mediating the cellular identity transition through its dynamic alterations regulating pluripotency maintenance and early lineage differentiation, so these markers do not specifically apply to our study.

## 9. Conclusions

In summary, phyllodes tumors represent a unique and complex category of fibroepithelial neoplasms that require careful clinical and histopathological evaluation for accurate diagnosis and management. The rarity of these tumors, coupled with their unpredictable biological behavior, necessitates a comprehensive understanding of their epidemiology, clinical presentation, histopathological features, and treatment strategies. Ongoing research into the molecular and genetic characteristics of phyllodes tumors may provide valuable insights into their pathogenesis and inform future therapeutic approaches. As the medical community continues to grapple with the challenges posed by phyllodes tumors, a concerted effort to standardize diagnostic criteria and treatment protocols will be essential in improving patient outcomes and reducing the risk of recurrence. Ultimately, enhancing awareness and understanding of phyllodes tumors among healthcare providers will contribute to better management and support for patients affected by this rare condition.

## Figures and Tables

**Table 1:** Overview of tumor dimensions in different studies.

Author		Yamada	Lin	Zhou		Di Liso	Jagdewsing
MD (R) (cm)	B	4.9	6 (1.5–28.0)	<5 129 (77.7)	≥5 37 (22.3)	25.5 ± 18.5 (4–130)	5.58 ± 2.29
	BL	7.5		115 (65.7)	60 (34.3)	34.7 ± 24.4 (10–110)	10.58 ± 6.79
	M	7.5		21 (43.8)	27 (56.3)	57.4 ± 60.2 (18–250)	14.90 ± 6.44

**Table 2:** Surgical treatment, recurrences and survival rate in several studies.

Author		Yamada	Lin	Zhou	Di Liso	Jagdewsing
Surgical treatment/ histology	Conservative surgery	110/110B	26/8B + 13BL + 5M	428/B + BL	77/54B + 23BL	77/54B + 23BL
	Mastectomy	13/8BL + 5M	7/7M	26/26M	24/13BL + 11M	24/13BL + 11M
Recurrence	B	6	1	6	6	6
	BL	0	3	26	2	2
	M	2	5	22	3	3
Overall outcome (5-year overall survival)		47 month survival	81.0%	88.4%	83.6%	Not evaluated

## Data Availability

No new data were created or analyzed in this study. Data sharing is not applicable to this article.

## Abbreviations

The following abbreviations are used in this manuscript:

ADC	Apparent diffusion coefficient
B	Benign
BC	Breast carcinoma
CT	Computed tomography
BL	Borderline
DWI	Diffusion-weighted imaging
ER	Estrogen receptors
FA	Fibroadenoma
HPF	High-power field
IHC	Immunohistochemical Markers
M	Malignant
MRI	Magnetic resonance imaging
MD	Mean dimensions
OS	Overall survival
PR	Progesterone receptor
R	Range
PT	Phyllodes tumor
SMA	Smooth muscle actin
US	Ultrasound
VEGF	Vascular endothelial growth factor
WHO	World Health Organization

## References

[1] Tan P.H. (2021). Fibroepithelial lesions revisited: Implications for diagnosis and management. Mod. Pathol..

[2] Lissidini G., Mulè A., Santoro A., Papa G., Nicosia L., Cassano E., Ashoor A.A., Veronesi P., Pantanowitz L., Hornick J.L. (2022). Malignant phyllodes tumor of the breast: A systematic review. Pathologica.

[3] Chen J.J., Zhu I., Patel A., Krings G., Chen Y.Y., Yuen F., Mukhtar R.A., Melisko M., Singer L., Park C.C. (2024). Management of Concurrent M Phyllodes Tumor and Invasive Breast Carcinoma. Adv. Radiat. Oncol..

[4] Di Liso E., Bottosso M., Lo Mele M., Tsvetkova V., Dieci M.V., Miglietta F., Falci C., Faggioni G., Tasca G., Giorgi C.A. (2020). Prognostic factors in phyllodes tumours of the breast: Retrospective study on 166 consecutive cases. ESMO Open.

[5] Li X., Jiang N., Zhang C., Luo X., Zhong P., Fang J. (2021). Value of conventional magnetic resonance imaging texture analysis in the differential diagnosis of benign and borderline/malignant phyllodes tumors of the breast. Cancer Imaging.

[6] Lerwill M.F., Lee A.H.S., Tan P.H. (2022). Fibroepithelial tumours of the breast—A review. Virchows Arch..

[7] Zhou Z.R., Wang C.C., Sun X.J., Yang Z.Z., Chen X.X., Shao Z.M., Yu X.L., Guo X.M. (2018). Prognostic factors in breast phyllodes tumors: A nomogram based on a retrospective cohort study of 404 patients. Cancer Med..

[8] Khatiwada A., Bastakoti A., Kc S., Sharma U., Rao S.M. (2024). Transformation of recurrent benign phyllodes tumor: A case report and comprehensive review of literature. Ann. Med. Surg..

[9] Hamdy O., Saleh G.A., Raafat S., Shebl A.M., Denewer A. (2019). Male Breast Huge Malignant Phyllodes. Chirurgia.

[10] Yom C.K. (2021). Malignant Phyllodes of Breast. Adv. Exp. Med. Biol..

[11] Fujimoto A., Matsuura K., Hasebe T., Saeki T. (2021). Phyllodes tumour arising in the ectopic axillary breast tissue, mimicking axillary lymphadenopathy. BMJ Case Rep..

[12] Maciulaitis T., Rimdeikaite M., Gudaviciene D., Jakutis N. (2025). Giant juvenile phyllodes tumour: A case report. Front. Surg..

[13] Yu C.Y., Huang T.W., Tam K.W. (2022). Management of phyllodes tumor: A systematic review and meta-analysis of real-world evidence. Int. J. Surg..

[14] Jagdewsing D.R., Murtaza G., Jagdewsing S.A., Jagdewsing S.A., Fahmy N.S.C., Silva F.A., Koendjbiharie T., Djojomoenawi S., Kwakye O.V., Mahmud N.M. (2024). Evaluation of the Clinicopathological Features Associated With Malignancy of Phyllodes Tumor of the Breast. Int. J. Clin. Oncol..

[15] Lu Y., Chen Y., Zhu L., Cartwright P., Song E., Jacobs L., Chen K. (2019). Local Recurrence of Benign, Borderline, and Malignant Phyllodes Tumors of the Breast: A Systematic Review and Meta-analysis. Ann. Surg. Oncol..

[16] Lin C.C., Chang H.W., Lin C.Y., Chiu C.F., Yeh S.P. (2013). The clinical features and prognosis of phyllodes tumors: A single institution experience in Taiwan. Int. J. Clin. Oncol..

[17] Rajgopal V., Sammader S., Ranjan P., Mohammed F. (2022). Phyllodes tumour presenting in a rare location: A case report and literature review. Int. Surg. J..

[18] Mustață L., Gică N., Botezatu R., Chirculescu R., Gică C., Peltecu G., Panaitescu A.M. (2022). Malignant Phyllodes Tumor of the Breast and Pregnancy: A Rare Case Report and Literature Review. Medicina.

[19] Li X., Chai W., Sun K., Fu C., Yan F. (2022). The value of whole-tumor histogram and texture analysis based on apparent diffusion coefficient (ADC) maps for the discrimination of breast fibroepithelial lesions: Corresponds to clinical management decisions. Jpn. J. Radiol..

[20] Shibuya M. (2013). Vascular endothelial growth factor and its receptor system: Physiological functions in angiogenesis and pathological roles in various diseases. J. Biochem..

[21] Torres L.A.F., Celso D.S.G., Defante M.L.R., Alzogaray V., Bearse M., de Melo Lopes A.C.F.M. (2024). Ki-67 as a marker for differentiating BL and benign phyllodes tumors of the breast: A meta-analysis and systematic review. Ann. Diagn. Pathol..

[22] Li J.J., Tse G.M. (2020). Core needle biopsy diagnosis of fibroepithelial lesions of the breast: A diagnostic challenge. Pathology.

[23] Zhang L., Yang C., Pfeifer J.D., Caprioli R.M., Judd A.M., Patterson N.H., Reyzer M.L., Norris J.L., Maluf H.M. (2020). Histopathologic, immunophenotypic, and proteomics characteristics of low-grade phyllodes tumor and fibroadenoma: More similarities than differences. Breast Cancer.

[24] Hasan A., Mohammed Y., Basiony M., Hanbazazh M., Samman A., Abdelaleem M.F., Nasr M., Abozeid H., Mohamed H.I., Faisal M. (2023). Clinico-pathological features and immunohistochemical comparison of p16, p53, and Ki-67 expression in muscle-invasive and non-muscle-invasive conventional urothelial bladder carcinoma. Clin. Pract..

[25] Rakha E., Mihai R., Abbas A., Bennett R., Campora M., Morena P., Toss M., Ellis I. (2021). Diagnostic concordance of phyllodes tumour of the breast. Histopathology.

[26] Ye J., Theparee T., Bean G.R., Rutland C.D., Schwartz C.J., Vohra P., Allard G., Wang A., Hosfield E.M., Peng Y. (2024). Targeted DNA Sequencing in Diagnosis of M Phyllodes Tumors With Emphasis on Tumors With Keratin and p63 Expression. Mod. Pathol..

[27] Kim S., Kim J.-Y., Kim D.H., Jung W.H., Koo J.S. (2013). Analysis of phyllodes tumor recurrence according to the histologic grade. Breast Cancer Res. Treat..

[28] Cervoni G.E., Quintana L., Erlinger A.L., Neo D.T., Recht A., Schnitt S.J., Hacker M.R., Sharma R. (2019). Local recurrence after breast- conserving therapy for phyllodes tumors: A 15-year retrospective review. Breast J..

[29] Lim R.S., Cordeiro E., Lau J., Lim A., Roberts A., Seely J. (2021). Phyllodes tumors-the predictors and detection of recurrence. Can. Assoc. Radiol. J..

[30] Tukenmez M., Mollavelioglu B., Onder S., Emiroglu S., Velidedeoglu M., Ergun S., Cabioglu N., Muslumanoglu M. (2023). Surgery for phyllodes tumour of the breast. What should be surgical margins?. ANZ J. Surg..

[31] Saxena P., Lalchandani A., Dausage C. (2020). Recurrent phyllodes tumour of breast infiltrating the latissimus dorsi reconstruction flap. BMJ Case Rep..

[32] Gupta K., Puri G., Kataria K., Jayaram J. (2022). Complex chest wall reconstruction after excision of malignant phyllodes tumour. BMJ Case Rep..

[33] Chao X., Chen K., Zeng J., Bi Z., Guo M., Chen Y., Yao Y., Wu W., Shi L., Nie Y. (2019). Adjuvant radiotherapy and chemotherapy for patients with breast phyllodes tumors: A systematic review and meta-analysis. BMC Cancer.

[34] Roberts A.C., Lunt L.G., Coogan A.C., Madrigrano A. (2023). The Role of Radiation Therapy in Locally Advanced Breast Cancer in a Patient With Li-Fraumeni Syndrome. Am. Surg..

[35] Amir R.A., Rabah R.S., Sheikh S.S. (2018). Malignant phyllodes tumor of the breast with metastasis to the pancreas: A case report and review of literature. Case Rep. Oncol. Med..

[36] Wakankar R., Dharmashaktu Y., Venugopal A., Kumar R. (2025). Malignant Phyllodes Tumor With Sphenoid Bone Metastasis Detected on 99m Tc-MDP SPECT/CT and 18 F-FDG PET/CT. Clin. Nucl. Med..

[37] Ostapenko E., Burneckis A., Ostapenko A., Skaisgirytė A., Ostapenko V. (2022). M phyllodes tumor of the breast with metastases to the lungs: A case report and literature review. Radiol. Case Rep..

[38] Zieba D., Pories S., Thota H.B., Suster D.I. (2025). Malignant Phyllodes Tumor of the Breast With Multiple Cutaneous Metastasis Resembling Pleomorphic Rhabdomyosarcoma. Am. J. Dermatopathol..

[39] Charoenyothakun A., Shotelersuk K., Nantavithya C., Saksornchai K. (2025). The impact of adjuvant radiotherapy on borderline and malignant phyllodes tumors of the breast. Breast Cancer.

[40] Pezner R.D., Schultheiss T.E., Paz I.B. (2008). Malignant Phyllodes Tumor of the Breast: Local Control Rates With Surgery Alone. Int. J. Radiat. Oncol..

[41] Pandey M., Mathew A., K J., Abraham E.K., Mathew B.S., Rajan B., Nair K.M. (2001). Malignant Phyllodes Tumor. Breast J..

[42] Valenza C., Trapani D., Porta F.M., Olmeda E., Gaeta A., Boscolo Bielo L., Conversano F., De Pas T.M., Castellano G., Santoro C. (2025). The pathologic and genomic evolution of primary malignant phyllodes tumors of the breast: Retrospective cohort study and case-control genomic analysis. Oncologist.

[43] Slachmuylders E., Laenen A., Vernemmen A., Keupers M., Nevelsteen I., Han S.N., Neven P., Van Ongeval C., Wildiers H., Smeets A. (2025). Expression patterns of H3K27me3 for differentiation of breast fibroadenomas and phyllodes tumors. APMIS Acta Pathol. Microbiol. Immunol. Scand..

